# Saturable plasmonic metasurfaces for laser mode locking

**DOI:** 10.1038/s41377-020-0291-2

**Published:** 2020-03-31

**Authors:** Jiyong Wang, Aurelien Coillet, Olivier Demichel, Zhiqiang Wang, Davi Rego, Alexandre Bouhelier, Philippe Grelu, Benoit Cluzel

**Affiliations:** 1 Laboratoire Interdisciplinaire Carnot de Bourgogne, Université Bourgogne Franche-Comté, 9 avenue Alain Savary, 21078 Dijon, France; 2 Key Laboratory of 3D Micro/Nano Fabrication and Characterization of Zhejiang Province, School of Engineering, Westlake University, 18 Shilongshan Road, 310024 Hangzhou, Zhejiang Province China; 3 Institute of Advanced Technology, Westlake Institute for Advanced Study, 18 Shilongshan Road, 310024 Hangzhou, Zhejiang Province China; 4 Department of Electrotechnology, Federal Institute of Bahia, R. Emídio dos Santos, 40301015 Salvador, Brazil

**Keywords:** Optical materials and structures, Optical techniques, Lasers, LEDs and light sources

## Abstract

Metamaterials are artificial materials made of subwavelength elementary cells that give rise to unexpected wave properties that do not exist naturally. However, these properties are generally achieved due to 3D patterning, which is hardly feasible at short wavelengths in the visible and near-infrared regions targeted by most photonic applications. To overcome this limitation, metasurfaces, which are the 2D counterparts of metamaterials, have emerged as promising platforms that are compatible with planar nanotechnologies and thus mass production, which platforms the properties of a metamaterial into a 2D sheet. In the linear regime, wavefront manipulation for lensing, holography, and polarization control has been achieved recently. Interest in metasurfaces operating in the nonlinear regime has also increased due to the ability of metasurfaces to efficiently convert incident light into harmonic frequencies with unusual polarization properties. However, to date, the nonlinear absorption of metasurfaces has been mostly ignored. Here, we demonstrate that plasmonic metasurfaces behave as saturable absorbers with modulation performances superior to the modulation performance of other 2D materials and exhibit unusual polarimetric nonlinear transfer functions. We quantify the link between saturable absorption, the plasmonic resonances of the unit cell and their distribution in a 2D metasurface, and finally provide a practical implementation by integrating the metasurfaces into a fiber laser cavity operating in pulsed regimes driven by the metasurface properties. As such, this work provides new perspectives on ultrathin nonlinear saturable absorbers for applications where tunable nonlinear transfer functions are needed, such as in ultrafast lasers or neuromorphic circuits.

## Metasurfaces: high quality mode locking

The demonstration that plasmonic metasurfaces can function as an ultrathin nonlinear saturable absorber brings new opportunities for modelocking lasers. Jiyong Wang and coworkers from France, China and Brazil designed and fabricated metasurfaces based on an array of gold nanorods, with a length of ~500nm, on glass. Experiments show that the metasurfaces act as an efficient saturable absorbers in the near-infrared with very strong modulation properties at a wavelength of ~1550 nm. When placed into a fibre laser cavity, the metasurface functioned as a modelocking element enabling the generation of a train of high quality ultrashort soliton pulses with sub-picosecond duration and a signal-to-noise ratio as high as 75 dB. The metasurfaces may also prove to be useful for realizing nonlinear transfer functions required for neuromorphic information processing.

## Introduction

Plasmonic metasurfaces are artificial 2D sheets of plasmonic unit cells that repeat in a subwavelength array^1–3^. In such metasurfaces, the plasmon resonance of the unit cell improves the interaction of the impinging light through an enhanced absorption cross-section^4–6^. Plasmon resonance allows metasurfaces to be much thinner than the wavelength, whereas the spatial distribution and orientation of the unit cell determine the amplitude and phase distribution of the light scattered in the far field^7^. Such properties, which originate from the generalized Snell–Descartes law, have paved the way toward the concept of flat optics, which aims to develop ultrathin sheets of metasurfaces mimicking the properties of traditional bulky optical elements^3,8,9^. Flat lenses^10^, polarizers^11,12^, holograms, and collimators^13,14^ to name a few have been successfully implemented for practical applications^15^. In parallel to these works on waveshaping, Moreau et al.^5^ evidenced that light absorption can also be greatly enhanced in plasmonic metasurfaces. In this field, quasi-perfect absorbers have been extensively studied due to their potential applications in filtering^16^, photosensing^17^, or photovoltaics^18^. However, from the perspective of practical applications, little attention has been paid to metasurfaces under pulsed laser illumination, in which optical nonlinearities become significant. To the best of our knowledge, frequency conversion effects in nonlinear metasurfaces, such as subwavelength hole arrays, split-ring resonators, and fishnet structures, have mainly been considered, and a remarkably high frequency conversion efficiency and unusual polarization properties have been reported^19^.

For 20 years, the nonlinear absorption of plasmonic devices has also attracted much interest as it leads to a complex cascade of electronic processes involving hot electrons, which are of particular interest for many applications^20–22^ ranging from photochemistry to ultrafast photodetectors^23,24^. Indeed, the nonlinear absorption of gold plasmonic devices leads to the excitation of a hot-electron cloud relaxing at a sub-picosecond timescale, which can be used to seed another physical or chemical process^25–28^. In this ultrafast electronic excitation regime, the reduction in the occupation of the electronic states well below the gold Fermi level when the electron distribution becomes athermal leads to transient absorption related to a change in the metal dielectric function^25,29^. It has finally been shown that gold absorption can be saturated under intense optical pumping^30^, which can be helpful for achieving pulsed regimes in laser architectures^31,32^. Indeed, to generate stable ultrashort pulses through mode locking, the laser cavity requires the action of a saturable absorber with an ultrafast relaxation time, a significant contrast in the nonlinear transfer function, and a high damage threshold^33–35^. Several saturable absorber 2D materials have been developed during the past two decades, aiming to achieve convenient integration in fiber laser and waveguide laser platforms. Single-walled carbon nanotubes^36^, graphene^37^, topological insulators^38^, and black phosphorus^39^ are among the most investigated low-dimensional materials in this respect, although their nonlinear contrast is limited at the level of a few percent. Considering the pioneering works using plasmonic nanoparticles as saturable absorbers in fiber lasers^31,32,34,35^, only colloidal solutions of gold nanoparticles with dispersed sizes and random orientations have been used. Therefore, the optical properties behave more like averaged properties or closer to the bulk material effects, and the conclusions drawn have been poorly linked to the plasmonic landscape.

In this work, we employ planar technologies to fabricate 2D plasmonic metasurfaces with a precisely defined size, gap, and orientation, and thus a well-controlled plasmonic mode that synthesized counterparts can barely handle. The nonlinear properties of such metasurfaces, specifically their saturable absorption when exposed to intense laser pumping, are systematically investigated. The link between the input polarization and the geometric parameters of plasmonic metasurfaces is highlighted. In particular, the efficiency of saturable metasurfaces for laser mode locking is further validated by integrating the metasurfaces in a fiber laser cavity architecture to promote stable self-starting soliton mode locking.

## Results

### Linear characterization

**Fig. 1:**
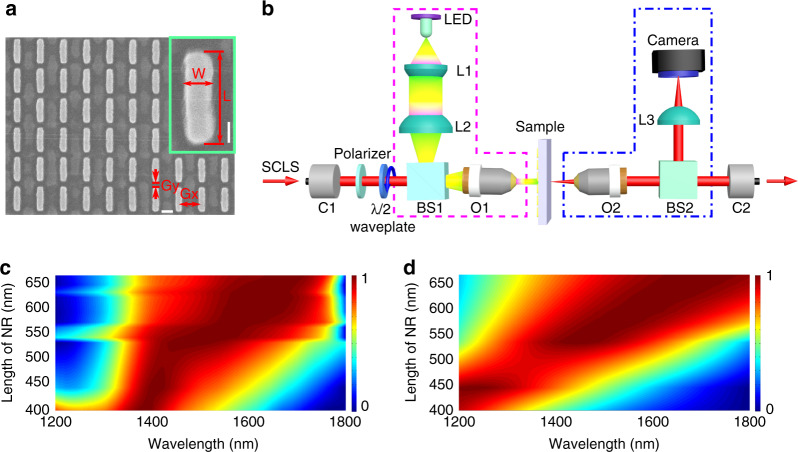
**a** SEM image of an NR array with a 50 nm gap in the long-axis direction (Gy) and a 300 nm gap in the short-axis direction (Gx). The horizontal scale bar represents 200 nm. The inset shows a single NR from this array, which has a length (L) of 445 nm and a width (W) of 120 nm. The vertical scale bar represents 100 nm. **b** Optical setup for measuring the extinction spectra of NR arrays, where SCLS is a supercontinuum light source, C1 and C2 are collimators, LED is a light-emitting diode, L1, L2, and L3 are lenses, BS1 and BS2 are beam splitters, and O1 and O2 are microscope objectives. The pink and blue dashed lines represent the illumination and imaging optical paths, respectively. **c**, **d** Normalized experimental and simulated extinction spectra of the NR arrays with different values of L

Plasmonic metasurfaces made of gold nanorods (NRs) for which plasmonic properties are well established are first designed and fabricated. In total, 50 µm by 50-µm arrays of NRs with various lengths are fabricated on glass slides using electron-beam lithography followed by evaporation of a 50-nm gold film and lift-off. The length (L) of a single NR is varied from 400 nm to 670 nm, with a width (W) of 120 nm for the two shortest lengths and 150 nm for the other lengths. A detailed description of the fabrication procedure can be found in the “Supporting Information”. All of the fabricated arrays are characterized by scanning electron microscopy (SEM) after all of the optical measurements are performed. Figure 1a shows an example of an NR array with L = 445 nm and W = 120 nm. The two adjacent NRs have a 50 nm gap in the long-axis direction (Gy), and a 300 nm gap in the short-axis direction (Gx). The inset shows a single NR in the array.

The linear properties of the plasmonic NRs are characterized in a home-built extinction microscope operating in the near-infrared region. As depicted in Fig. 1b, the sample is sandwiched between two confocal objectives (O1 and O2, NA of 0.3, focus diameter < 10 µm), which are conjugated on both sides with fiber optics collimators (C1 and C2). Köhler illumination is implemented with a visible LED on one side (pink dashed lines), and a CCD camera allows for sample visualization on the other side (blue dashed lines). To measure the transmission of the samples, a supercontinuum light source (SCLS) emitting in the 1–2 µm spectral range is focused on the sample and combined with the illumination using a beam splitter (BS1). A polarizer and a half-waveplate control the polarization axis of the incident SCLS, and a second beam splitter (BS2) separates the transmitted light for sample imaging and transmittance measurements. BS1 and BS2 are mounted on magnetic holders so that one can easily switch from imaging to optical measurements.

The extinction spectrum of the *i*th NR array, which denotes the fingerprint of plasmonic resonance, is defined as ${\mathrm{Ext}}_i\left( \lambda \right) = \left[ {T_{{\mathrm{ref}}}\left( \lambda \right) - T_{{\mathrm{nr}},i}\left( \lambda \right)} \right]/T_{{\mathrm{ref}}}\left( \lambda \right)$, where *T*_ref_(*λ*) is the transmission outside the NR array, *T*_nr,i_(*λ*) is the transmission of the *i*th NR array, and *λ* is the wavelength. Theoretical extinction spectra are calculated by using commercial finite-element software, taking the periodical condition into account. The numerical simulations show that the plasmonic landscape of the reported metasurfaces is mainly dominated by the dipolar resonances of the individual nanoantenna. Indeed, the role of periodicity remains very weak since the periods used here are sufficiently lower than the wavelength to prevent a far-field effect, but large enough to significantly reduce the near-field coupling between individual nanoantennas (see “Supporting Information” for the details)^40,41^. The experimental and theoretical extinction spectra of different NR arrays are shown in Fig. 1c, d for an incident polarization parallel to the NR axis. As can be seen from the comparison with the numerical extinction spectra in Fig. 1d, the calculations generally agree very well with the experimental results, apart from a slightly larger peak wavelength shift and broader spectral linewidths. More importantly, the experiments confirm that plasmonic resonances can be obtained at 1550 nm for NR lengths of ~500 nm, which allows for their nonlinear characterization using a standard laser and detectors at telecom wavelengths.

### Nonlinear characterization

**Fig. 2:**
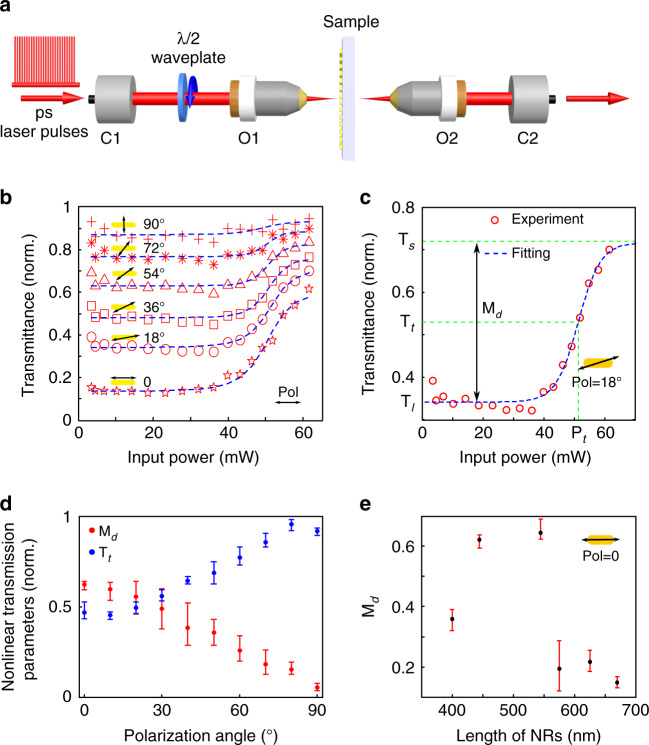
**a** Optical setup for measuring the nonlinear transmission of NR arrays. **b** The experimental transmission of the NR array as a function of the input power for different excitation polarizations (from 0° to 90° with steps of 18° with respect to the long axis of the NR). The experimental data (red symbols) are fitted by using a generalized sigmoid function (blue curves). **c** The experimental transmission (red circles) of the NR array as a function of the input power with an excitation polarization of 18° with respect to the long axis of the NR. The modulation depth M_*d*_ and typical transmission T_*t*_ are defined from the corresponding fittings (blue curve). The transmission of each NR array in (**a**–**c**) is normalized to the value of the nearby blank glass slide. **d** Experimental modulation depth M_*d*_ and typical transmission T_*t*_ as functions of the excitation polarization angle. **e** Experimental modulation depth M_*d*_ as a function of the length of NRs when the excitation polarization is along the long axis of the NRs. The error bars in (**d**) and (**e**) indicate the variations in M_*d*_ and T_*m*_ considering the polarization symmetry when the polarization angle has been rotated by a whole revolution (360°)

Following the linear optical characterization, the nonlinear transmittance of the plasmonic NR metasurfaces is recorded using the same setup mentioned previously, apart from the light source, as shown in Fig. 2a. To saturate the absorption of the NR array, we use a home-built pulsed laser with a pulse duration of 500 ps and a repetition rate of 100 kHz. The maximum average output power of the laser is 100 mW, corresponding to a peak power of 2 kW (see “Supporting Information” for details). The laser wavelength is 1555 nm, and its polarization is linear, such that a half-waveplate can be used to tune the polarization axis with respect to the NR orientation. Using this laser, we record the transmission of each NR array, and use the transmission of the nearby blank glass slide as a reference.

The experimental results of the transmission of an NR array with L = 445 nm are plotted in Fig. 2b for different polarization angles, ranging from 0° and 90° with respect to the long axis of the NRs. One can first note that the “S”-shaped profile of these curves clearly reveals the nonlinear transmission of the fabricated metasurfaces. At low power, the transmission of the NR array remains linear with a transmission coefficient T_*l*_, which depends on the polarization of the input laser. T_*l*_ is inversely proportional to the absorption cross-section of the NR, which becomes maximal when the incident polarization matches the long axis of the NRs^42^. Above a typical power P_*t*_, the transmission increases in a nonlinear fashion until it reaches a saturated value T_*s*_, showing the saturation of the metasurface absorption. The parameters P_*t*_, T_*l*_, and T_*s*_ are illustrated in Fig. 2c, which shows the nonlinear transmission for an incident polarization of 18°. P_*t*_ refers to the excitation power corresponding to the typical transmission level T_*t*_ = (T_*s*_ + T_*l*_)/2. Given the “S”-shaped profile, the experimental data are fitted using a sigmoid function (blue dashed lines in Fig. 2b, c), from which the parameters T_*l*_, T_*s*_, P_*t*_, and T_*t*_ used hereafter are extracted.

The typical transmission T_*t*_ and the modulation depth of the saturable absorption M_*d*_ = T_*s*_ − T_*l*_ strongly depend on the polarization of the input laser. Indeed, the transmittance of NRs perpendicular to the input light polarization remains high regardless of the input power, while strong absorption occurs due to collective plasmonic resonances when the input polarization matches the long axis of the NRs. In the latter case, the plasmonic absorption becomes saturated at high powers, and the resulting modulation depth can reach values as high as M_*d*_ = 60% as shown in Fig. 2d. Such high modulation depths are uncommon, especially for thin metasurfaces: a comparison between 2D saturable absorbers shows that the maximum modulation depth reported is less than 11%^43^, and a similar study with colloidal gold NRs reports an M_*d*_ of 5.46%^44^. For a comparison, a typical semiconductor saturable absorber mirror (SESAM) can achieve an M_*d*_ of 30%, but in a much thicker device^45^.

To cross-check the relationship between the plasmon resonances of the metasurfaces and the saturable absorption, the effect of the NR length on the modulation depth of the saturable absorption is also investigated. The results presented in Fig. 2e correspond to the case where the polarization of the incident light is parallel to the long axis of the NRs, for which the modulation depth is the highest. The measurements clearly show that the modulation depth reaches a maximum for lengths of ~500 nm, as the plasmonic resonance matches the laser wavelength used in the experiment. As such, this result strengthens the conclusion that the nonlinear absorption is directly linked to the plasmonic resonances of the NR arrays.

### Polarimetric saturable absorption

**Fig. 3:**
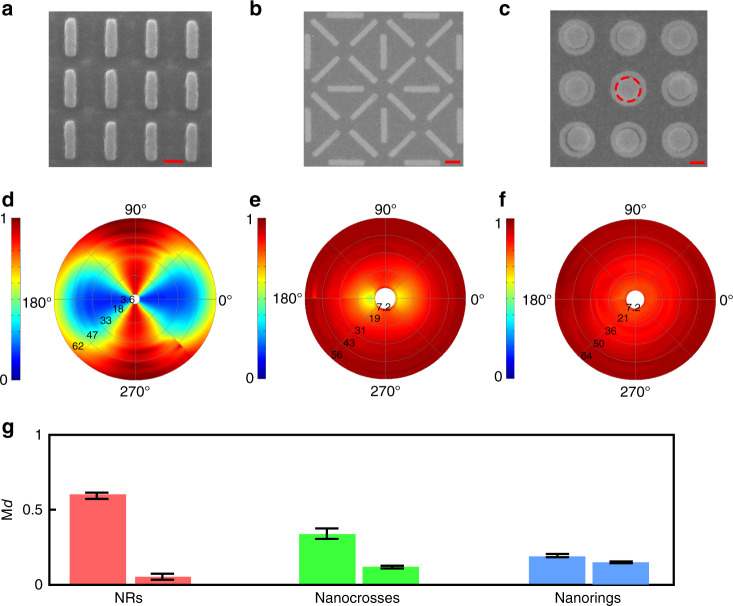
SEM images (**a**–**c**) and excitation power- and polarization-dependent nonlinear transmission (**d**–**f**) of an NR array (**a**, **d**), nanocross array (**b**, **e**), and nanoring array (**c**, **f**). The scale bars in the SEM images represent 200 nm. **g** Comparisons of the modulation depth M_*d*_ among the NRs (red bars), nanocrosses (green bars) and nanorings (blue bars). Two bars denote two extreme polarizations, where M_*d*_ has maximum (left bar) and minimum (right bar) values. The error bars indicate the variations in M_*d*_ by considering the polarization symmetry when the excitation polarization angle has been rotated by 360°

The previous results show a large dependence of the nonlinear absorption of the NR arrays on the input polarization. Due to the versatility of our approach, new structures referred to as “nanocrosses” and “nanorings” are designed and fabricated to change this polarization dependence. Figure 3b, c shows the SEM images of these two structures, arranged in arrays, similar to the previous case of the NRs shown in Fig. 3a.

Figure 3b shows four unit cells of nanocrosses, with each consisting of eight NRs. The long axes of the pairs of NRs are rotated by 0°, 90°, 45°, and −45° so that the polarization dependence is largely reduced compared with that of a single orientation NR array. All of the NRs have the same width of 90 nm and the same length of 480 nm, which corresponds to maximum absorption at ~1550 nm. Figure 3c shows the SEM image of nanorings, which have an inner diameter of 320 nm and an outer diameter of 500 nm. Here, we notice that the central parts of the nanorings, marked as dashed circles in the figure, are filled with conductive polymer (Electra 92 from ALLRESIST GmbH) for SEM observations. For such dimensions, the theoretical dipolar plasmon mode is calculated with a finite-element method, and a resonance is found at ~1550 nm (see “Supporting Information” for details). Due to the symmetry, the nanorings almost do not show any polarization-dependent resonances.

After the fabrication, the two samples of nanocrosses and nanorings are optically tested using the same setup presented earlier, both in the linear and nonlinear regimes. To better visualize the experimental behavior of the different structures, a polar pseudocolour representation is used, where the polar coordinates (*P, θ*) represent the input power and the input polarization, and the colormap represents the transmission level of the array. With this representation, the saturable absorption along the long axis of the NR array (Fig. 3d) clearly appears, while the polarization dependence is strongly reduced in the case of the nanocrosses (Fig. 3e) and almost entirely disappears for the nanorings (Fig. 3f). This reduction in the polarization dependence also occurs with a decrease in the modulation depth M_*d*_ to 33% at the maximum in the case of the nanocrosses and as low as 19% for the nanorings, as shown in Fig. 3g. These results show that one can tailor the polarization dependence of the nonlinear absorption of the metasurfaces but at the expense of the modulation depth, although a polarization-insensitive metasurface still displays remarkable saturable absorption features.

### Ultrafast fiber laser architecture

**Fig. 4:**
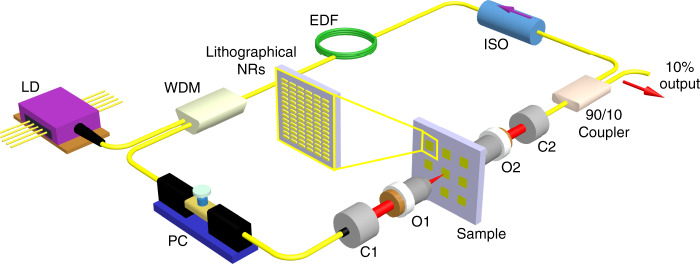
Scheme of a home-built ultrafast fiber laser that integrates lithographical NRs as a saturable absorber, where LD represents the laser diode, WDM is the wavelength-division multiplexer, EDF is the erbium-doped fiber, ISO is the optical isolator, PC is the polarization controller, C1 and C2 are the collimators, and O1 and O2 are the objectives

In order to further test the fabricated samples and provide a practical demonstration of the saturable metasurface application for ultrashort laser pulse generation, we finally integrated the metasurfaces into a fiber laser cavity. In a laser cavity, the saturable absorption of the plasmonic arrays is expected to lead to the formation of ultrashort pulses, ultimately reaching passively mode-locked regimes. To test whether the plasmonic metasurfaces shown earlier can act as the mode-locking element of a fiber laser, we build the fiber cavity shown in Fig. 4, which includes a 980 nm pump diode (LD, maximum pump power of 1 W), a 980/1550 nm wavelength multiplexer (WDM), 0.5 m of erbium-doped fiber (EDF), a polarization-insensitive isolator (ISO), a polarization controller (PC), and an output coupler. The free-space setup from Fig. 1b, without the polarizer and half-waveplate, completes the cavity, allowing for the integration of the NR arrays. Note that the sample has to be tilted at a slight angle, below 10°, to avoid back reflections that would perturb the normal operation of the laser. The overall length of the cavity is 7.5 m with anomalous net chromatic dispersion, and all of the fiber connections are made with standard single-mode silica fibers. The total loss of the plasmonic metasurfaces plus the free-space optical elements integrated in the laser cavity, as shown in Fig. 4, is usually below 3 dB, and can be as low as 2 dB.

The output of the laser is taken from the 10% output of the fiber coupler and sent to our detection setup, which includes a fast photodiode connected to a 6-GHz real-time oscilloscope or an electrical spectrum analyzer, a time-averaged optical spectrum analyzer (OSA), a multishot optical autocorrelator, and a real-time optical spectrum measurement line. The latter employs dispersive Fourier transform (DFT), which maps the spectrum of each output pulse onto a temporal waveform by stretching the laser output within a long dispersive fiber link^46,47^. Precisely, our DFT setup consists of a 6-km-long dispersion compensating fiber that provides a total accumulated dispersion of 686 ps/nm and a 14-GHz photodiode connected to the 6-GHz oscilloscope. This configuration therefore leads to an electronic-limited spectral resolution of 0.03 THz (0.24 nm).

**Fig. 5:**
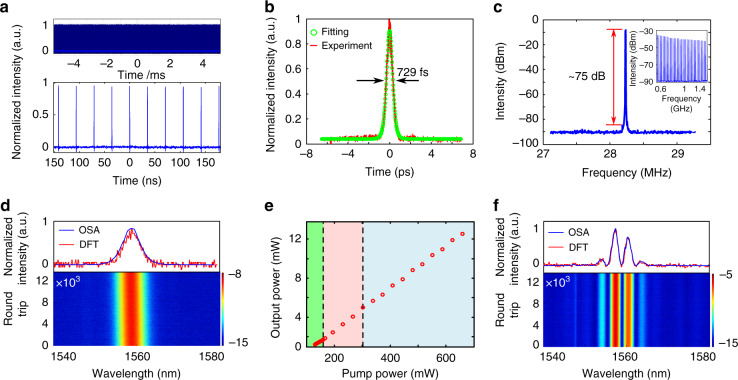
Plasmonic NR metasurfaces implemented in ultrafast laser architectures. **a** Pulse train shown on the oscilloscope over short (300ns, lower panel) and long (10ms, upper panel) time ranges. **b** Autocorrelation trace of the single soliton. **c** Radio-frequency optical spectrum at the fundamental frequency and wideband RF spectrum (inset). **d** Averaged optical spectrum from the OSA (blue) and single-shot spectrum from the DFT (red). The lower panel shows the evolution of the spectrum measured with the DFT setup over 12,000 roundtrips. **e** Laser output power as a function of pump power. The single-soliton regime (green background), single soliton with continuous-wave components (red background) and soliton molecule regime (blue background) appear sequentially with an increase in pump power. **f** Soliton molecule spectra recorded on the OSA (blue) and oscilloscope by using the DFT (red). The lower panel shows the evolution of the spectrum measured with the DFT setup over 12,000 roundtrips

When an NR array is placed in the fiber laser cavity, stable mode locking is achieved by increasing the pump power and tuning the polarization controller. It should be noted that the blank glass slide without the NR array does not lead to the generation of pulses within the cavity, only continuous-wave emission, regardless of the pumping power and polarization controller orientation. Thus, it unambiguously confirms the role of the plasmonic metasurface as the mode-locking element of the cavity. The characterization of a typical mode-locked regime achieved with the NR array is presented in Fig. 5. Figure 5a presents an oscilloscope time trace showing that a stable pulse train with a repetition rate of 28.2 MHz is achieved, in relationship with the fundamental roundtrip time of the 7.5-m-long fiber cavity. The intensity autocorrelation trace from Fig. 5b indicates that a single soliton is generated inside the cavity, and that its duration is 729 fs when fitted with a hyperbolic-secant profile. The pulse train stability is assessed by sending the photodetected signal to a radio-frequency spectrum analyzer, resulting in the measurements of Fig. 5c. The signal reveals a very pure signal at 28.2 MHz and its harmonics, with a large signal-to-noise ratio of 75 dB, which demonstrates the remarkably high stability of the pulse train for such a cavity laser scheme with some free-space elements^48^. The time-averaged pulse spectrum recorded on the OSA is shown in Fig. 5d, and compares well with a single-shot DFT spectrum. The central wavelength is located at 1558 nm, and the full-width at half-maximum (FWHM) of the spectrum is 5 nm. Compared with the Fourier transform-limited FWHM spectral width (3.5 nm for a hyperbolic-secant profile or 4.8 nm for a Gaussian profile), this result shows the generation of a pulse with a low amount of frequency chirping, which is a convenient feature for most applications. Using a long recording of the DFT signal, we can follow the evolution of the spectrum over a large number of successive roundtrips, 12,000 in this case, as shown in the lower part of Fig. 5d. The virtually unchanged shape of the spectrum once again confirms the very high stability of the mode-locked regime.

When the pump power is increased, the laser output goes through several regimes: at first, the laser operates in a quasi-continuous-wave regime, with only a few spectral lines. When the pump power increases sufficiently such that the random fluctuations of the intensity within the laser cavity approach the threshold power P_*t*_ of the saturable absorber, the highest fluctuation is amplified and shaped into an ultrashort pulse within a few hundred roundtrips. At the laser output, the soliton pulse has a fixed duration and peak power, which depends on the cavity dispersive and dissipative parameters that globally determine a dissipative soliton attractor^49^. In the present cavity architecture, the peak power is mostly fixed by the saturable absorber features, whereas the pulse duration is approximately determined by the interplay between the anomalous cavity dispersion and the Kerr nonlinearity (i.e., soliton pulse shaping). Therefore, a further increase in the pump power will hardly change the pulse energy. Rather, most of the additional energy introduced in the cavity transfers into the build-up of dispersive waves and a quasi-continuous background until eventually a second, identical, soliton is generated. These steps are summarized in Fig. 5e with the corresponding output power from the mode-locked laser. In the high-pumping regime where two solitons coexist in the laser cavity, these two solitons interact to form a soliton molecule. Soliton molecules are ubiquitous in ultrafast laser dynamics^47^ and consist of two solitons stably bound at a close distance, generally of a few pulse widths, by virtue of a specific attractor^49^. The time interval between both pulses, which is 2.4 ps, is inferred from the high-contrast interference pattern appearing in the optical spectrum featuring a 3.3 nm period, as seen in Fig. 5f. The stability of the observed soliton molecule is also remarkable. It is also worth noting that no visible thermal damage is observed from such NR saturable absorbers during ultrafast pulse generation in the mode-locked regime even at the maximal pump power (1 W), indicating that plasmonic metasurfaces indeed exhibit good thermal conductivity and stability against optical damage (see “Supporting Information” for details).

**Fig. 6:**
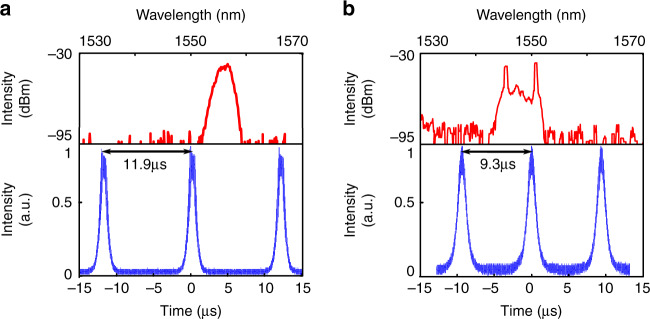
The Q-switching laser pulse characterization by using nanocrosses (**a**) and nanorings (**b**) as SAs

If the NR array is replaced by either a nanocross or a nanoring array, we do not obtain stable mode-locked operation, such as in the previous case, but we obtain periodic bursts of pulses, as shown in the oscilloscope traces of Fig. 6a, b, respectively. This Q-switching operation, which is weakly dependent on the polarization controller orientation, has a period in the microsecond range, corresponding to the relaxation oscillations of the gain medium. Compared with an NR array, this change in behavior can be explained either by the lower modulation depth M_*d*_ of the metasurface or by the strong polarization dependence of the NRs. Indeed, the latter is expected to assist the saturable absorber action of the NRs in the array configuration through the nonlinear evolution of the polarization that occurs in the optical fibers^50^.

## Discussion

In summary, we demonstrate here that plasmonic gold metasurfaces are saturable absorbers with ultrahigh modulation performances and unusual polarization properties that can be fully controlled by the design. In particular, we quantify the link between gold saturable absorption, the plasmonic resonances of the unit cell and their distribution in a 2D metasurface. Finally, a practical implementation of the saturable metasurfaces is demonstrated by integrating the fabricated metasurfaces into a fiber laser cavity, which leads to the generation of pulse trains. In the case of NR arrays inserted in the laser cavity, we obtain highly stable mode-locked regimes generating either ultrashort soliton or soliton molecules. Compared with the properties of other 2D or semiconductor materials, the remarkable properties reported here mainly originate from the plasmonic nature of the metasurfaces themselves. Indeed, metasurfaces combine the high extinction coefficient of gold, which exceeds the extinction coefficient of other materials by at least one to two orders of magnitude, plasmonic resonances, whose wavelength and polarimetric properties can be tuned by design, and the carrier relaxation dynamics in gold, which lie on the sub-picosecond scale. It is also expected that the saturable metasurfaces reported here could benefit from recent developments in epsilon-near-zero materials to further increase the modulation depth and reduce its saturation threshold^51^. As such, saturable plasmonic metasurfaces offer all of the ingredients and degrees of freedom needed to design novel ultrathin and efficient saturable absorbers with unusual polarimetric properties that could find applications where complex nonlinear transfer functions are needed, such as in ultrafast laser architectures or neuromorphic circuits.

## Materials and methods

All of the details about the nanofabrication, extinction simulations, home-built nanosecond pulsed laser, and damage threshold tests on the plasmonic metasurfaces are provided in the Supplementary Material.

## Supplementary information

Supplemental materials
