# Supplementary material for: Saturable plasmonic metasurfaces for laser mode locking
Source: Light Sci Appl. 2020 Mar 31;9:50. doi: 10.1038/s41377-020-0291-2 (PMC7109045; doi:10.1038/s41377-020-0291-2)
Supplement: Supplementary file 1 — Supplemental materials [file 41377_2020_291_MOESM1_ESM.docx]

Supporting Information

Saturable plasmonic metasurfaces for laser mode-locking

*Jiyong Wang,^1,2,3^Aurelien Coillet,^1^Olivier Demichel,^1^Zhiqiang Wang,^1^Davi Rego,^1,4^Alexandre Bouhelier,^1^ Philippe Grelu^1^ and Benoit Cluzel^1^**

^1^Laboratoire Interdisciplinaire Carnot de Bourgogne, Université de Bourgogne Franche-Comté, 9 avenue Alain Savary, 21078 Dijon, France

^2^Key Laboratory of 3D Micro/Nano Fabrication and Characterization of Zhejiang Province, School of Engineering, Westlake University, 18 Shilongshan Road, 310024 Hangzhou, Zhejiang Province, China

^3^Institute of Advanced Technology, Westlake Institute for Advanced Study, 18 Shilongshan Road, 310024 Hangzhou, Zhejiang Province, China

^4^Department of Electrotechnology, Federal Institute of Bahia, R. Emídio dos Santos, 40301015 Salvador, Brazil

*Corresponding author

Benoit Cluzel, E-mail: [benoit.cluzel@u-bourgogne.fr](mailto:benoit.cluzel@u-bourgogne.fr), Telephone: +33 380 39 60 10;

1. Nano fabrication

After cleaning by piranha solution in an ultrasonic bath, the substrate (150 μm thick glass cover slip) was coated with the first layer of electron-sensitive resist (Poly-Methyl-Metacrylate (PMMA) 50 K) in a spin-coater (spinning speed: 5000 rpm, acceleration speed: 3000 rpm s-1, duration: 60 s). After soft baking (hot plate at 150 °C for 3 minutes), the substrate was spin-coated (spinning speed: 4000 rpm, acceleration speed: 3000 rpm s-1, duration: 60 s) with a second 200K-PMMA layer. After the second soft baking, we spin-coated a thin layer of conductive polymer (Electra 92 from ALLRESIST GmbH) to prevent charge accumulation during the exposure. Then the resist was exposed with the pre-designed patterns by the electron beam. After the conductive polymer was removed in water, the written patterns were developed via a developer (AR 600-56 from ALLRESIST) and diluted with stopper (IPA). In the development, the exposed areas of the resist were removed. Then a physical vapor deposition (PVD) process was used to coat the sample with a 2 nm thick Cr layer (for adhesion purposes) and a 50 nm thick Au layer. The sample was finally immersed in acetone for more than 3 hours to lift-off the remainder of the resist.

2.Extinction simulation

In order to simulate the extinction spectra in according with the experimental conditions, a finite elements method is used. In the model system, as shown in Figure S1, the NR is located on a substrate of refractive index n*_b_* = 1.5, excited by a polarized plane waves coming from the air side (n*_a_*=1) of the interface. The angle of incidence is θ = 90°. The geometric parameters used for the computation derived from the SEM images of the NR arrays, which are W=2R=120 nm with a length of 400 nm and 445 nm, and W = 150 nm for the NRs left. In order to take into account the grating effects in a nanostructure array, the periodic conditions in both x and y directions are applied. The total extinction spectra are calculated by: Extinction(j,λ)=1-T(j,λ), where j is the number of NR arrays, λ is the excitation wavelength, T is the total transmission when the incident light passes through the periodic NR array.


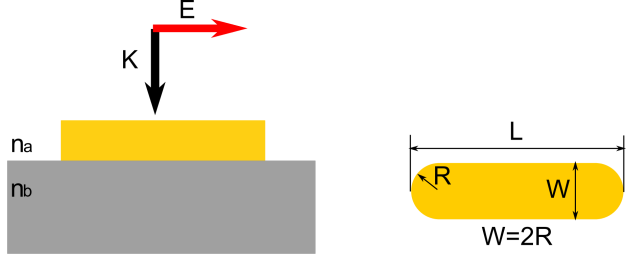


**Figure S1.** NR modeled by using a finite element method

3. Home-built nano-second pulse laser


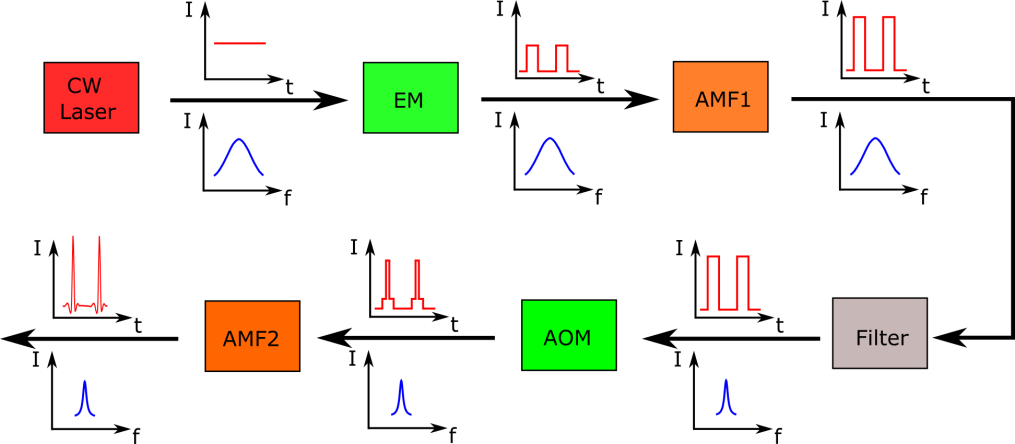


**Figure S2.** Schematic diagram of a home-built nanosecond pulse laser, where CW is continuous wavelength, EM is electrical modulator, AMF1and AMF2 are amplifiers, AOM is acousto-optic modulator.

A home built ps pulse laser is employed for the nonlinear absorption/transmission experiment. As can be seen from Figure S2, a CW laser beam (central wavelength: 1550 nm) is firstly modulated into 500-ps pulses by an electro-optic modulator, followed by a first stage of optical amplification (AMF1). The beam then passes through a tunable band-pass filter, so that the amplified spontaneous emission of the first amplifier is mostly eliminated. Because of the 20 dB extinction ratio of the electro-optic modulator, a continuous-wave background remains in the optical signal. The repetition rate being of 100 kHz, most of the energy at this stage correspond to this CW background. An acousto-optic modulator with an extinction ratio of 40 dB and driven by a 60-ns pulse voltage generator is therefore used to remove most of this continuous wave. The obtained pulses are further amplified by the second optical amplifier so that the output averaged power can be adjusted from 0 to 1W.

4. Extinction spectra of nanorings

By using the same method as described above-“Extinction simulation“, the nanorods are replaced with the nanorings, as shown in Figure S3(a).The extinction spectra for the nanorings array with different inner diameters *d* is shown in Figure S3(b). The nanorings keep the constant wall thickness *T* of 90 nm. The white dashed line indicates the wavelength of 1555 nm.


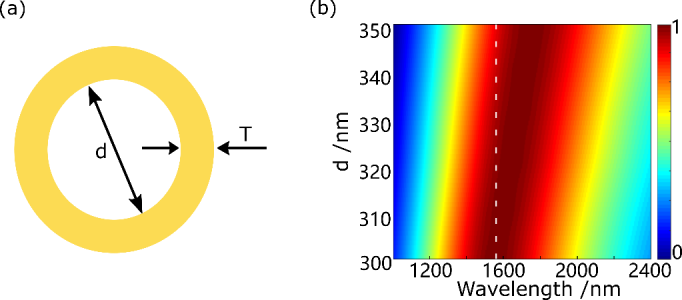


**Figure S3.** Normalized simulated extinction spectra of the nanoring arrays

**5. Damage threshold test on the plasmonic metasurfaces**

The thermal damage tests were conducted during our nonlinear characterization from 9 nanorod, 9 nanocross and 9 nanoring samples. As an example shown in Figure S4, the thermal damages were observed once the input power was beyond around 60 mW. Given 3 dB of optical loss in the whole setups and 50% percent of absorption, the averaged power for the metasurfaces is around 15 mW. By using our home-built ps pulse laser (pulse duration 500 ps, repetition rate 100 KHz, see more details in 3^rd^ part of SI), for a input power of 60 mW, the estimated energy per pulse is around 100 nJ pulse-1 which corresponds to a damage threshold in the beam waist (slightly smaller than10 µm as visible on the SEM picture) larger than 0.19 J cm-2.





**Figure S4.** SEM image of thermal damage test for the nanocross metasurfaces

From the literature [J. Phys. Chem. B 2000, 104, 6152-6163.] and [J. Opt. Soc. Am. B 1996, 13(2), 459-468.], the damage threshold of gold nanorods is within a 0.1-1 J cm-2 range which is in the same range of our value but it largely depends on the pulse duration, repetition rate and the wavelength which were not comparable to the experiments reported here.

When the mode-locked pulses (pulse duration 729 fs, repetition rate 28.2 MHz) were generated, the maximum averaged power in our fiber laser cavity was 25 mW (when the pump power reaches to its maximum 1W). The maximum laser fluence inside the laser cavity can be calculated as 1.1mJ cm-2, which is much lower than the damage threshold reported in the literature above. Therefore, no visible thermal damages were observed from SEM images after all the mode-locking experiments.
